# IL-15 Harnesses Pro-inflammatory Function of TEMRA CD8 in Kidney-Transplant Recipients

**DOI:** 10.3389/fimmu.2017.00778

**Published:** 2017-06-30

**Authors:** Gaëlle Tilly, Tra-My Doan-Ngoc, Michelle Yap, Aurélie Caristan, Lola Jacquemont, Richard Danger, Marion Cadoux, Sarah Bruneau, Magali Giral, Pierrick Guerif, Bryan Nicol, Alexandra Garcia, David-Axel Laplaud, Sophie Brouard, Claire Pecqueur Hellman, Nicolas Degauque

**Affiliations:** ^1^Centre de Recherche en Transplantation et Immunologie UMR1064, INSERM, Université de Nantes, Nantes, France; ^2^Institut de Transplantation Urologie Néphrologie (ITUN), CHU Nantes, Nantes, France; ^3^CRCNA UMR 892/CNRS 6299, INSERM, Université de Nantes, Nantes, France

**Keywords:** TEMRA CD8, kidney transplantation, immunometabolism, IL-15, inflammatory cytokines, glycolysis and oxidative phosphorylation, CD8 T cells

## Abstract

The involvement of TEMRA CD8 is evident in a large array of immunological conditions ranging from auto- to allo-immunity. Nevertheless, the factors leading to their accumulation and activation remain ill-defined and, efficient therapeutics to control their inflammatory response is lacking. Here, we show that IL-15-stimulated TEMRA from kidney-transplant (KT) recipients promote inflammation by inducing the expression of CX3CL1 by endothelial cells in an IFN-γ- and TNF-α-dependent manner. The responsiveness of TEMRA to IL-15 is not restricted to chronic stimulation, as TEMRA from healthy volunteers respond earlier and faster when compared to effector memory (EM). IL-15 induces antiapoptotic signals and promotes proliferation dependent of PI3K/Akt, MAPK, and ERK pathways. Without *ex vivo* stimulation, TEMRA cells are metabolically more active than naive and EM, as shown by their high ATP reservoir and a high expression of genes involved in glycolysis, glutaminolysis, and the Pentose Phosphate Pathway. Upon stimulation, TEMRA adapt their metabolism by sustaining an increased mitochondrial respiration and glycolysis. Finally, we show that the inhibition of glycolysis is highly effective in preventing endothelial inflammation induced by TEMRA from KT recipients. Together, our findings highlight the metabolic fitness that tightly regulates the immune function of TEMRA in physiological and pathogenic situations.

## Introduction

Expansion of pathogenic CD8 effector memory (EM) lymphocytes that re-express CD45RA (TEMRA) have been evidenced in a large array of diseases from autoimmunity [multiple sclerosis (1), lupus (2)] to allo-transplantation (3) and bone regeneration (4). For instance, we recently reported that the expansion of TEMRA CD8 can be detected in kidney-transplant (KT) recipients despite a long-term stable graft function and that such expansion is associated with a twofold higher risk of kidney graft dysfunction (3). These reports challenge the classical view that TEMRA CD8 accumulate with age or chronic antigen stimulation and as such lead to the immune system senescence (5–7).

The immune settings able to induce the activation and the expansion of TEMRA CD8 remain ill-defined and preclude the design of innovative strategies to control pathogenic TEMRA CD8. A recent report has suggested that IL-15 could promote cell-cycle entry of TEMRA and favor their migration in non-lymphoid tissues (8). IL-15 is a well-studied cytokine that is critical for the maintenance of memory CD8, including the control of their basal proliferation (9, 10), their trafficking (11), and their optimal response (12). However, IL-15 regulation of the immune function of *ex vivo* TEMRA purified from healthy volunteers (HV) or from KT has not been established. This prompts the need to investigate the effect of IL-15 stimulation on TEMRA CD8 functions, including mapping the signaling cascade to effector function, and looking into the effect of chronic alloantigen stimulation on TEMRA CD8 derived from KT. Being able to properly stimulate TEMRA CD8 response will enable the screening of new therapeutic strategies to control their pathogenicity, one strategy being a selective targeting of metabolic processes.

The ability to control the immune response by interfering with metabolic pathways has been successfully tested in various animal models including lupus (13), tumor vaccination (14), hematopoietic stem cell transplantation (15, 16), and heart and skin transplantation (17). The bioenergetic profiles have been primarily performed by comparing the properties of NAIVE CD8 and EM CD8 in human settings and in rodents. Metabolic reprogramming of memory CD8 accounts for their ability to rapidly respond to second stimulation (18, 19). For instance, memory CD8 has a greater mitochondrial mass which allows for a rapid metabolic response involving oxidative phosphorylation and aerobic glycolysis (18). Ligation of TCR on EM CD8 induces a rapid and sustained glycolytic switch that precedes clonal expansion (19). Very few reports have characterized the metabolic profiles of TEMRA CD8 (20, 21), and none have interrogate the regulation of their metabolism by IL-15.

In this study, we show that, despite immunosuppressive therapies, TEMRA CD8 from KT respond vigorously to IL-15 stimulation and foster the endothelium inflammation as shown by the upregulation of CX3CL1 on human umbilical vein endothelial cells (HUVECs) through the secretion of IFN-γ and TNF-α. The responsiveness of TEMRA CD8 to IL-15 stimulation is not restricted to pathogenic settings as a rapid upregulation of activation markers (CD25 and CD69) on TEMRA CD8 purified from HV is observed. Ligation of IL-15 to its receptor on TEMRA CD8 delivers pro-survival signals through the phosphorylation of Bad and pro-proliferative signals dependent on p38MAPK, ERK1/2, and PI3K/Akt pathways. We also demonstrate the metabolic fitness of TEMRA to rapidly respond to stimulation with a large pool of preformed ATP and the adaptation of their metabolism to stimulation with an increase in extracellular acidification rate (ECAR) and oxygen consumption rate (OCR). Finally, we show that the activation of endothelial inflammation by TEMRA CD8 from KT can be efficiently controlled by interfering with glycolysis and glutaminolysis processes.

## Materials and Methods

### Subjects and Ethics Statement

Peripheral blood mononuclear cells (PBMCs) were collected from HV and 56 KT (Table 1). All subjects gave written informed consent in accordance with the Declaration of Helsinki. HV were enrolled by the Etablissement Français du Sang (EFS, Nantes, France) within the context of a research contract. A convention has been signed between our laboratory (CRTI—INSERM UMR 1064) and the blood bank (Etablissement Français du Sang Pays de La Loire) and approval of an ethical committee was thus not necessary. The University Hospital Ethical Committee and the Committee for the Protection of Patients from Biological Risks approved the study for patients. The biological samples and data are gathered in accordance with French Law, more specifically with “Bioethical law” of August 6, 2004, Act no. 78-17 of January 6, 1978, on data processing, data, files, and individual liberties, with the European regulation: Directive 2004/23/EC of European Parliament and of the council of March 31, 2004 on setting, standards of quality and safety of donation, procurement, testing, processing, preservation, storage, and distribution of human tissue and cells, and with Directive 95/46/EC on the protection of individuals with regard to the processing of personal data and on the free movement of such data.

**Table 1:** Summary of demographic and clinical characteristics of patients.

		Mean	SD
At blood sampling	Recipient age (years)	49.16	11.07
	Donor age (years)	49.00	13.00
	Time-posttransplantation (years)	0.99	0.07
	Creatinemia (μmol/L)—3 months	149	57
	Creatinemia (μmol/L)—12 months	137	42
	Donor creatinemia (μmol/L)	136	189

		**Number**	**Percentage**

	Male recipient	23	74
	Male donor	25	81
	Incompatibility HLA-A,-B,-DR ≥ 4	6	19
Induction therapy	Polyclonal Ab	3	10
	Monoclonal Ab	28	90
Maintenance therapy	MMF	31	100
	AZA	0	0
	CNI	30	97
	mTOR inhibitor	1	3
	Corticotherapy	6	19
	Belatacept	0	0
CMV serology (donor/recipient)	D^−^/R^−^	27	48
	D^−^/R^+^	8	14
	D^+^/R^−^	10	18
	D^+^/R^+^	11	20

### Blood Samples

Peripheral blood mononuclear cells were separated on a Ficoll gradient layer and frozen in DMSO-10% autologous serum.

### Antibodies and Reagent

Antibodies against the following proteins were purchased from BD Biosciences: CD3 (HIT3a and UCHT1), CD69 (SK7), CD25 (M-A251), CD28 (CD28.2), pSTAT5 pY694 (clone 47), p38MAPK pT180/pY182 (38/p38), TNFα (Mab11), IFN-γ (B27), and Annexin V. Antibodies against the following proteins were purchased from Miltenyi Biotech: CD8 (BW135/80), CD45RA (T6D11), CCR7 (REA108), pAKT pS473 (REA359), and pERK1/2 pT202/pY204 (REA152). Antibodies against the following proteins were purchased from Cell Signaling Technology: pBAD S112 (40A9), p70 S6K pT389 (D57.2.2E), and p4EBP1 pT37/46 (236B4). IL-15 was purchased from Miltenyi Biotech. 2-Deoxy-d-glucose (2-DG), 6-Diazo-5-oxo-l-norleucine (DON), oligomycin, PD98059, SB203580, and LY294002 were obtained from Sigma. Granzyme B inhibitor IV was obtained from Calbiochem.

### Cell Culture and Flow Cytometry

TEMRA, NAIVE, and EM CD8 T cells were FACS-sorted according to the expression of CD45RA and CD28 using an ARIA cell-sorter flow cytometer. Highly purified CD8 T cell subsets were plated in 96-U bottom plates and cultured for 1–5 days in TexMACS medium (Miltenyi) with IL-15 (10 ng/mL; Miltenyi) and, when mentioned, coated anti-CD3 (OKT3; 2 µg/mL). Proliferation was monitored using the dilution of Cell Proliferation Dye eFluor450 (eBioscience) and assessed according to the frequency of CPD^low^ cells. After cell culture, apoptosis (Annexin V), cellular activation (CD25, CD69), and cell death (uptake of DAPI) were assessed. Interferences of glycolysis, glutaminolysis, and OXPHOS were performed by adding 2-DG (10 mM), DON (100 µM), and oligomycin (1.5 µM), respectively. PD98059, SB203580, and LY294002 were used at a final concentration of 10 μM. Cells were analyzed with a LSRII flow cytometer (BD Immunocytometry Systems). BD CompBeads stained separately with individual mAbs were used to define the compensation matrix. Data were analyzed using FlowJo Version 9.7.6 (TreeStar).

### Coculture of HUVEC and CD8 T Cells

Primary cultures of HUVECs were grown in Endothelial Cell Growth Medium 2 (LONZA). 1 × 10^5^ purified CD8 T from KT recipients were stimulated overnight with IL-15 (10 ng/mL) and coated anti-CD3 (2 µg/mL). Supernatant was harvested, and stimulated CD8 T cells were washed. 50 μL of supernatant or 10^5^ stimulated CD8 T cells were added to overnight deprived HUVEC seeded in 24-well plate (250 µL). Blocking antibodies anti-TNFα (10 µg/mL) and anti-IFN-γ (10 µg/mL) and Granzyme B inhibitor IV (10 µg/mL) were added to HUVEC–CD8 coculture. After 6 h of stimulation, HUVEC were stored in RLT Buffer (Qiagen).

### 2-NBDG Uptake

Purified CD8 subsets were cultured for 16 h, washed, and cultured for 2 h in glucose-free medium containing 50 µM 2-NBDG (Invitrogen) prior to analysis by flow cytometry.

### Quantification of Mitochondria and Mitochondrial Membrane Potential (MMP) Assessment

MitoTracker Green and MitoTracker Red were used to quantify and to assess the MMP, respectively. PBMCs were first incubated with 100 nM MitoTracker Green or MitoTracker Red (Invitrogen) for 30′ at 37°C 5% CO_2_ and then stained using anti-CD3, CD8, CD45RA, and CD28 antibodies. Samples were immediately analyzed with a LSRII flow cytometer.

### Phosphorylation of Signaling Molecules

Phosphorylation of STAT5 was measured using the protocol developed by Goldeck et al. (22). Phosphorylation of the other proteins was analyzed using the BD Phosflow Protocol. PBMCs were incubated in TexMACS at 37°C/5% CO_2_ for 2 h, washed once at RT, and the final concentration was adjusted to 1 × 10^7^ cells/mL. 10^6^ PBMCs were incubated in a final volume of 100 µL in a 96-U bottom plate for 30′ at 37°C/5% CO_2_. Pre-warmed IL-15 was added at a final concentration of 10 ng/mL for 15′ at 37°C/5% CO_2_. Reaction was rapidly stopped by transferring the plate on ice and the addition of 100 µL of cold FACS buffer (pSTAT5) followed by the permeabilization and fixation with Cytofix/Cytoperm buffer (BD Biosciences) or by adding an equal volume of pre-warmed Cytofix Buffer for 10′ at 37°C/5% CO_2_ (other Phosflow Antibody). PBMCs were stained for cell-surface markers in Perm/Wash buffer for 30′ at 4°C before (pSTAT5) or after (other Phosflow Antibody) being further permeabilized by adding cold BD Perm Buffer III. Stability of the staining of cell-surface markers upon the use of BD Perm Buffer III was ensured in preliminary experiments.

### Quantification of ATP

Purified CD8 T subsets cells were incubated in TexMacs at 4 × 10^6^ cells/mL and stimulated for 45′ to 24 h various time with plate-bound anti-CD3 (2 µg/mL) and IL-15 (10 ng/mL). 15 µL of each cells suspension (i.e., 6 × 10e^4^ cells) were used for luminometric ATP measurement using Apo Biovision kit according to manufacturer’s instructions (Clinisciences). Luminometry was measured with a VICTOR multilabel plate reader (Perkin Elmer).

### Metabolic Assays

Oxygen consumption rate and ECAR were measured using Seahorse XF24 or XF96 analyzers in purified CD8 T cell subsets (4 × 10^5^ or 2.5 × 10^5^ purified cells, respectively) that were allowed to rest overnight after FACS-sorting in TexMACS buffer at 37°C/5% CO_2_. The assay was performed in Seahorse XF-base medium supplemented with 10 mM glucose (Sigma), 2 mM glutamine (Life Technologies), and 1 mM pyruvate (Life Technologies). Mitochondrial stress assay was performed by adding successively oligomycin (1.5 µM; Sigma), CCCP (1 µM; Sigma), and Antimycin A + Rotenone (1 µM each; Sigma). To assess OCR and ECAR upon polyclonal stimulation, PMA (50 ng/mL; Sigma) and Ionomycin (500 ng/mL; Sigma) were added 75′ after the start of the experiment. 2-DG (250 mM; Sigma) and oligomycin (1.5 µM; Sigma) were added before the stimulation with PMA-Iono.

### Real-time Quantitative PCR

RNA were extracted using RNeasy Micro Kit (Qiagen), and total RNA was reverse-transcribed using a classical MMLV cDNA synthesis (Invitrogen). Quantitative real-time PCR was performed using a ViiA 7 Real-Time PCR System (Applied Biosystems), and all TaqMan primer-probe sets were purchased as “Assay-on-Demand” from Applied Biosystems. References of primer-probe sets are available upon request. Transcript levels were calculated according to the 2^−ΔCt^ method as described by Applied Biosystems and normalized to the expression of 18S except for HUVEC based assay (GAPDH).

### TCR Repertoire Analysis

Qualitative analysis of the TCR Vβ repertoire was performed using previously described procedures (1, 23, 24). Briefly, CDR3 was amplified by PCR in a Veriti Thermal Cycler (Applied Biosystems), in separate reactions using a common reverse Cβ primer and different forward Vβ-specific primers (1). Amplicons generated after two semi-nested PCRs were labeled using a FAM-tagged Cβ primer, and the CDR3-length distribution (CDR3-LD) was determined with an AB3037 DNA sequencer (Applied Biosystems) and analyzed using GeneMapper software (Applied Biosystems). A software was developed to automatically describe individual TCR Vβ profiles and to compare the TCR Vβ usage across different samples. As previously described (1), four typologies of CDR3-LD were observed: monoclonal, oligoclonal, polyclonal with major peak, and polyclonal. A *correlation coefficient* (1) and a *distance score* (1) were used to compare TCR Vβ usage between CD8 subsets and pool of human thymus as reference profiles of CDR3-LD of each TCR Vβ family. Highly similar profiles are characterized by a high correlation coefficient and a low-distance score. The correlation coefficient and the distance score were very effective at distinguishing close or very different distributions, but not those in between.

### Statistical Analyses

Statistics were analyzed using Graphpad Prism. Mann–Whitney *U* test, Kruskal–Wallis test followed by Dunn’s *post hoc* test and paired Wilcoxon test were used when suitable and the nature of the test is mentioned within the legend figures. Exact *p*-values are mentioned or using * (*p < 0.05; **p < 0.01; ***p < 0.001; ****p < 0.0001).

## Results

### IL-15-Stimulated TEMRA CD8 from KT Recipients Induces the Activation of Endothelium through IFN-γ and TNF-α Secretion

**Figure 1:**
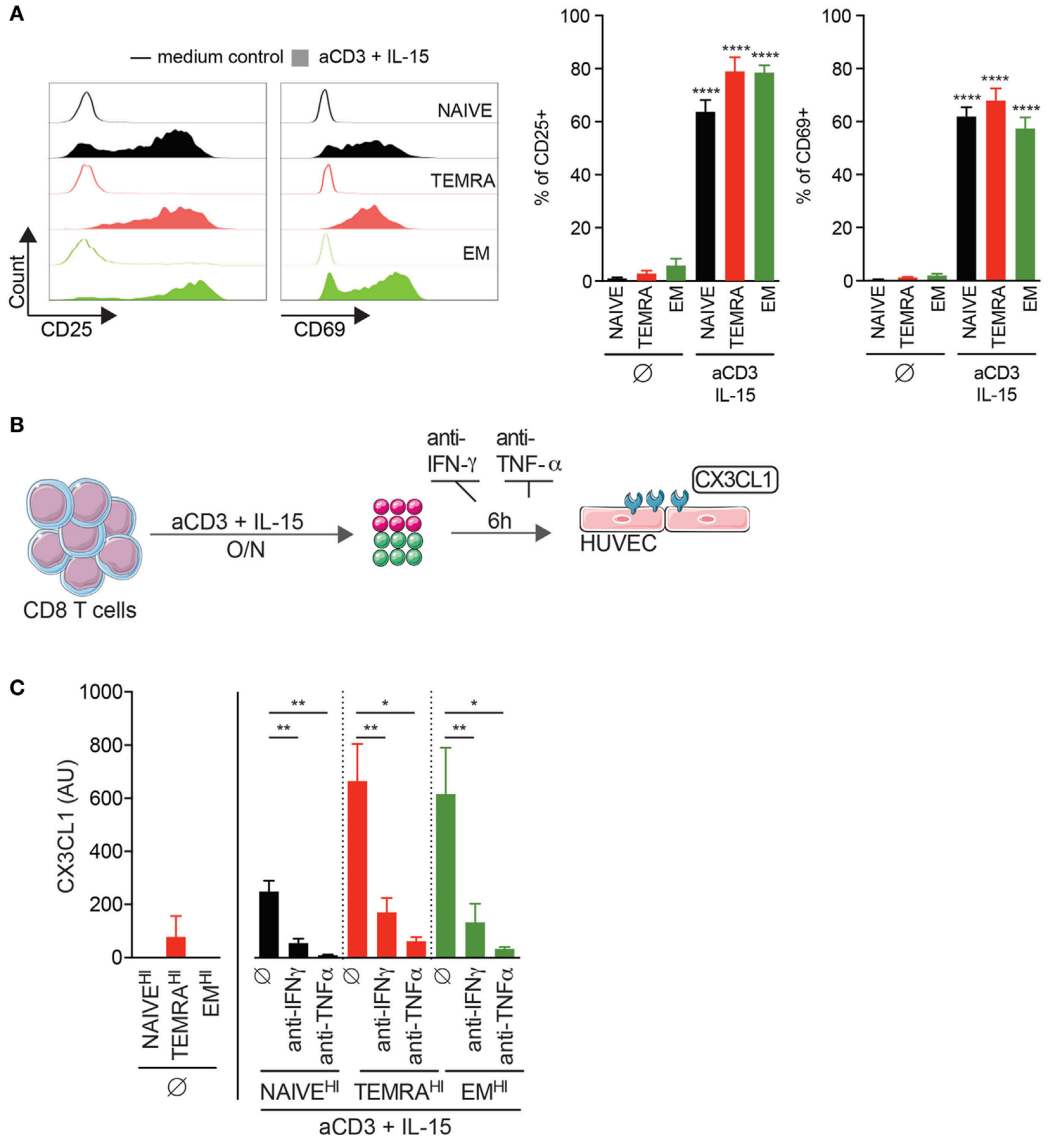
IL-15-stimulated TEMRA CD8 from Kidney-Transplant (KT) Recipients induces the activation of endothelium through IFN-γ and TNF-α secretion. **(A)** Expression of CD25 or CD69 by CD8 T cell subsets purified from KT recipients stimulated for 24 h with plate-bound anti-CD3 (2 µg/mL) and IL-15 (10 ng/mL). Representative flow data are shown, and bars indicate mean ± SEM of pooled data (*n* = 10–12). **(B)** Schematic overview of the strategy to assess the inflammation of human umbilical vein endothelial cell (HUVEC) by IL-15-activated CD8 T cell subsets from KT. **(C)** Expression of CX3CL1 by HUVEC after 6 h of culture in the presence of medium control or selective inhibitors [anti-IFN-γ (10 µg/mL) or anti-TNF-γ (10 µg/mL) mAb] and with supernatant (dilution of supernatant of 1/6) from CD8 T cell subsets from KT stimulated overnight with plate-bound anti-CD3 (2 µg/mL) and IL-15 (10 ng/mL). Bars indicate mean ± SEM of pooled data (*n* = 4–6). Mann–Whitney test (A) or Kruskal–Wallis test followed by a Dunn’s Multiple Comparison Test **(C)** was performed (*p < 0.05, **p < 0.01, ****p < 0.0001).

We have recently demonstrated that an accumulation of TEMRA CD8 T cells was deleterious for long-term graft outcome (3) and were detected despite a stable graft function and the uptake of immunosuppressive drugs. Given the low expression of costimulatory molecules CD28 and CD27 by TEMRA CD8 T cells (25), we hypothesize that the effector functions of TEMRA CD8 T cells are triggered by pro-inflammatory cytokines such as IL-15, which are expressed and transpresented by conventional dendritic cells and macrophages (26, 27). Of note, CD45RA and CD28 markers were used to identify NAÏVE (CD45RA^+^CD28^+^), EM (CD45RA^−^CD28^+^), and TEMRA (CD45RA^+^CD28^−^) CD8 subsets (Figure S1 in Supplementary Material) (25). Stimulation with plate-bound anti-CD3 and IL-15 results in the upregulation of the activation marker CD25 and early proliferation marker CD69 by TEMRA CD8 from KT recipients (mean ± SEM 79.0 ± 5.4 and 67.8 ± 4.6%, respectively; *p* < 0.0001 vs. no stimulation; Figure 1A). Activation of the endothelium is a key step in the process leading to kidney graft rejection and CD8 T cells contribute to the activation of the endothelium through the secretion of pro-inflammatory cytokines (IFN-γ, TNF-α) and cytotoxic molecules (GZM-b). CD8 T cells were purified from KT recipients that exhibited three distinct profiles of CD8 subsets (namely NAIVE^HI^, TEMRA^HI^, and EM^HI^, with more than 50% of the aforementioned CD8 subsets) and activated overnight with plate-bound anti-CD3 and IL-15 (Figures 1B,C). Supernatants were added onto HUVEC for 6 h, and the expression of CX3CL1 by HUVEC was quantified by qRT-PCR. Of note, the expression of CX3CL1 was undetectable in unstimulated HUVEC and in HUVEC cocultured with the supernatant from unstimulated CD8 T cells (data not shown and Figure 1C). Supernatant from IL-15-stimulated TEMRA^HI^ and EM^HI^ CD8 T cells from KT recipients induce a high expression of CX3CL1 by HUVEC, whereas IL-15-stimulated NAIVE^HI^ CD8 triggered only a small expression of CX3CL1 (*p* < 0.05 vs. no stimulation; Figure 1C). Provision of blocking antibodies against IFN-γ or TNF-α prevented HUVEC activation (*p* < 0.05 vs. stimulation; Figure 1C), demonstrating that secretion of pro-inflammatory cytokines by TEMRA^HI^ and EM^HI^ CD8 T cells from KT recipients lead to the inflammation of endothelial cells. Collectively, we demonstrated that IL-15 is a potent inducer of pathogenic function of TEMRA CD8 T cells from KT recipients and that TEMRA CD8 contributes to the deleterious inflammation of the endothelium.

### IL-15 Delivers Pro-Survival and Proliferating Signals to TEMRA CD8 Dependent of PI3K/Akt, p38 MAPK, and pERK1/2 Pathways

**Figure 2:**
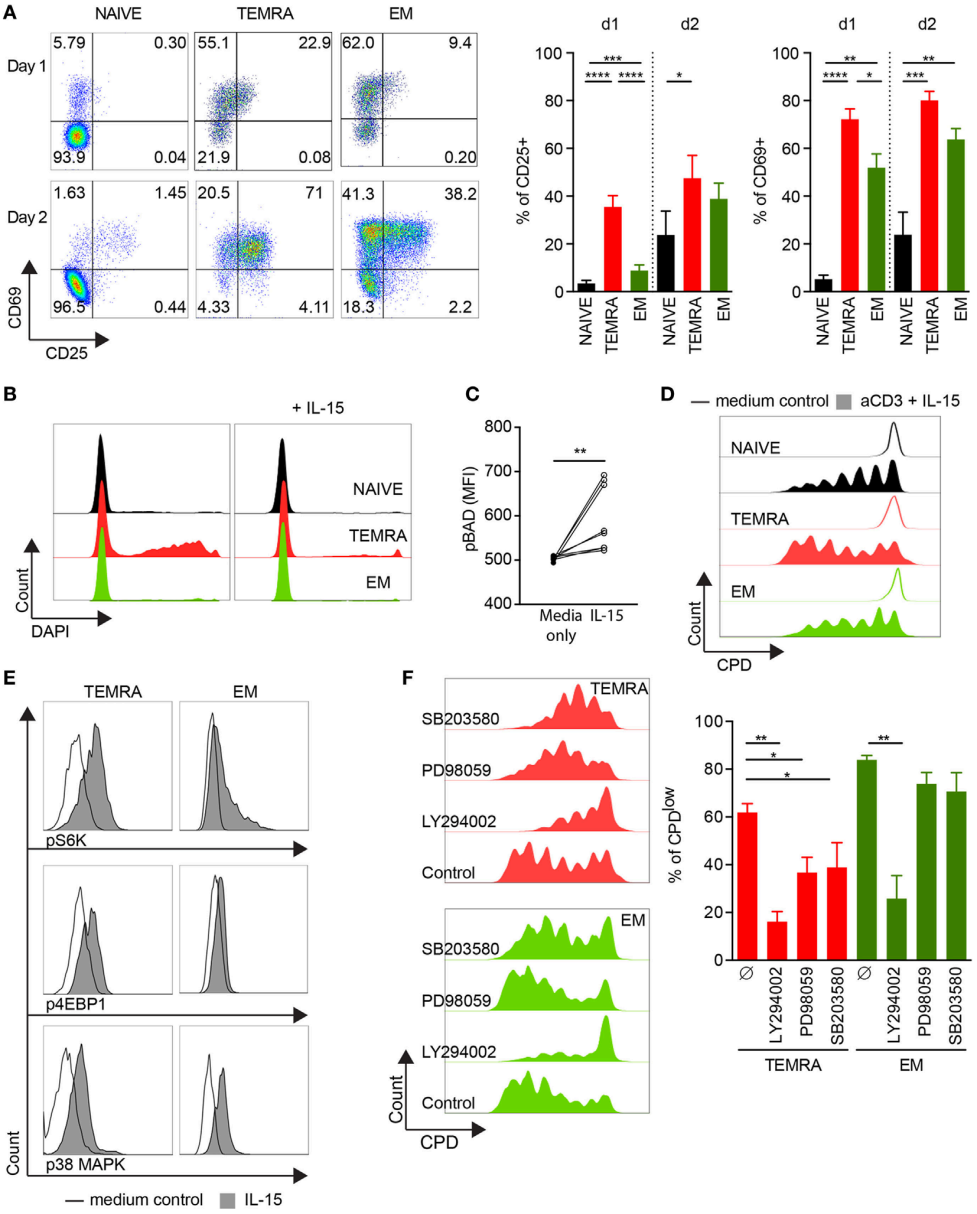
IL-15 delivers pro-survival and proliferating signals to TEMRA CD8 dependent of PI3K/Akt, p38 MAPK, and pERK1/2 pathways. **(A)**. Expression of CD25 and CD69 after 24 and 48 h of culture of purified CD8 subsets with plate-bound anti-CD3 (2 µg/mL) and IL-15 (10 ng/mL). Of note, neither CD25 nor CD69 could be detected on purified CD8 T cell subsets before anti-CD3 activation (data not shown). Representative flow data are shown, and bars indicate mean ± SEM of pooled data (*n* = 7–10). **(B)** Incorporation of DAPI after 48 h of culture of purified CD8 subsets with medium control (left panel) or with IL-15 (10 ng/mL; right panel). Representative flow data are shown (*n* = 9). **(C)** Peripheral blood mononuclear cells (PBMCs) were stimulated for 15′ with IL-15 (10 ng/mL) and phosphorylation of Bad (pS112) was analyzed within TEMRA CD8 T cells according to the mean fluorescent intensity. Each dot represents data obtained from cells isolated from one healthy volunteers (*n* = 8). **(D)** CPDeFluor405-labeled CD8 subsets were cultured for 5 days with plate-bound anti-CD3 (2 µg/mL) and IL-15 (10 ng/mL). Representative flow data are shown (*n* = 6). **(E)** PBMCs were stimulated for 15′ with IL-15 (10 ng/mL) or medium control and phosphorylation of p70 S6 Kinase (pT389), 4E-BP1 (pT37/46), and p-p38 MAPK (pT180/pY182) was analyzed within NAIVE, TEMRA, and effector memory (EM) CD8 T cells. Representative flow data are shown (*n* = 8). **(F)** Percentage of CPDeFluor405^low^ cells after 5 days of culture of purified CD8 subsets with plate-bound anti-CD3 (2 µg/mL) and IL-15 (10 ng/mL) in the presence of medium control or selective inhibitors (LY294002, 10 µM; PD98059, 10 µM; SB203580, 10 µM). Representative flow data are shown and bars indicate mean ± SEM of pooled data (*n* = 5–7). Comparison between the three CD8 subsets was performed using Kruskal–Wallis test followed by a Dunn’s Multiple Comparison Test **(A,F)**. *Post hoc* test was performed using the no-stimulation setting as reference for each CD8 subset **(A, F)** *p < 0.05, **p < 0.01, ***p < 0.001. Wilcoxon matched-pairs signed rank test was used to test the cytokine effect (C, no-stimulation setting as reference) *p < 0.05, **p < 0.01, ***p < 0.001, ****p < 0.0001.

The chronic stimulation of the immune system induced by allogeneic transplantation may alter the response of TEMRA CD8 T cells to IL-15. To test this hypothesis, we investigate the response of TEMRA CD8 T cells from HV to IL-15 stimulation. First, we investigated the upregulation of CD25 and CD69 (Figure 2A). CD8 subsets were FACS-sorted and cultured for 24 and 48 h with IL-15 in the presence of coated anti-CD3. IL-15 stimulation resulted in a rapid upregulation of CD25 and CD69 on TEMRA CD8 T cells, which can be observed as early as 24 h post-stimulation (mean ± SEM 35.5 ± 4.7 and 72.2 ± 4.4%, respectively; *p* < 0.0001 vs. no stimulation; Figure 2A). The activation kinetic of NAIVE and EM T cells is delayed as CD25 upregulation was observed only after 2 days. Despite the acquisition of CD69 by TEMRA and EM cells as early as day 1, the level of expression was greater on TEMRA as compared to EM (Figure 2A). Of note, neither CD25 nor CD69 could be detected on purified CD8 T cell subsets before anti-CD3 activation (data not shown). The regulation of TEMRA effector function by IL-15 was further investigated through the expression of pro-inflammatory cytokines (*IFN-*γ and *TNF-*α) and cytotoxic molecules (*GZMb* and *PERF*). Resting TEMRA CD8 cells highly express the T-box transcription factor T-bet which is associated with a high expression of *IFN-*γ, *TNF-*α, *GZMb*, and *PERF* (Figure S2A in Supplementary Material). Combined stimulation of TCR and IL-15 results in further enhancement of effector function of TEMRA CD8, characterized by an increase in *IFN-*γ and *TNF-*α after 48 h of stimulation (Figure S2B in Supplementary Material). Similar observations were made when EM CD8 were stimulated with TCR and IL-15 (Figure S2C in Supplementary Material). Collectively, these results demonstrate that the response to IL-15 stimulation of TEMRA CD8 was similar between HV and KT recipients.

As TEMRA CD8 T cells have been shown to exhibit a lower viability *in vitro* (20), we investigated whether IL-15 stimulation could rescue TEMRA CD8 cells from cell death. Purified CD8 T cells were cultured for 2 days in the presence of IL-15 only, and cell death was monitored according to the uptake of DAPI. As expected, in the absence of any stimulus, the frequency of DAPI^+^ cells was high in TEMRA CD8 T cells. However, the provision of IL-15 was sufficient to rescue TEMRA CD8 cells from cell death leading to a survival rate of TEMRA similar to those of NAÏVE and EM cells (Figure 2B). The inactivation of Bad (pS112), pro-apoptotic member of the Bcl-2 family, induced by IL-15 accounts for the pro-survival effect of IL-15 in TEMRA CD8 (*p* < 0.01; Figure 2C). Finally, we show that the activation of TEMRA CD8 cells by IL-15 is sustained for several days as a potent proliferation was observed after 5 days of stimulation with IL-15 and TCR with similar dilution of Cell Proliferation Dye across the 3 CD8 subsets (Figure 2D). Of note, TCR stimulation alone or IL-15 alone failed to induce significant proliferation of CD8 subsets (data not shown). Collectively, these results show that IL-15 triggers the potent effector function of TEMRA CD8 T cells.

A key characteristic of TEMRA CD8 in KT recipients is their highly restricted TCR Vβ repertoire (28). Further reinforcing the hypothesis that TEMRA CD8 in HV are similar to those in KT recipients, we compared the TCR Vβ repertoire usage between TEMRA, NAÏVE, and EM CD8 T cells purified from HV (Figure S3 in Supplementary Material). Whereas virtually all TCR Vβ families in NAÏVE CD8 exhibit a polyclonal distribution of their CDR3 length [TCR Vβ with polyclonal distribution of CDR3 length (CDR3-LD) mean ± SEM 91.8% ± 3.3], most of the TCR Vβ families in TEMRA CD8 exhibit preferentially few (PAPM profiles of CDR3-LD mean ± SEM 62.0% ± 5.6) to high selection (monoclonal profiles of CDR3-LD mean ± SEM 13.4% ± 4.4) of specific length of CDR3 (Figure S3A in Supplementary Material). TCR Vβ families with monoclonal CDR3-LD were significantly lower in EM CD8 T cells (monoclonal profiles of CDR3-LD mean ± SEM 0.9% ± 0.8). To confirm the accumulation of highly selected TCR Vβ families in TEMRA CD8, the profiles of CDR3 length obtained in purified CD8 subsets were compared to those obtained in a commercially available pool of thymocytes, sample used as prototypic profiles of absence of selection of any given CDR3 length. The profiles of TCR Vβ families in NAÏVE CD8 T cells were highly similar to the reference profiles (mean ± SEM 0.90 ± 0.01 and 30.2 ± 1.4 for correlation coefficient and distance score, respectively; Figures S3B,C in Supplementary Material). In contrast, the profiles of TCR Vβ families in TEMRA CD8 T cells were the most different (mean ± SEM 0.47 ± 0.02 and 100.5 ± 2.8 for correlation coefficient and distance score, respectively; Figures S3B,C in Supplementary Material). As compared to TEMRA CD8, the accumulation of selected clones in EM was lower (mean ± SEM 0.69 ± 0.02 and 66.9 ± 2.4 for correlation coefficient and distance score, respectively; Figures S3B,C in Supplementary Material). Collectively, these data show that the accumulation of selected clones in TEMRA CD8 T cells is shared between HV and KT recipients.

To gain further insight into the proliferation signaling pathways triggered by IL-15, we examined the role of p38 MAPK, ERK1/2, and AKT pathways. After 15′ of stimulation, resting TEMRA CD8 cells exhibited a potent phosphorylation of STAT5 in response to IL-15 (mean ± SEM 81.8% ± 4.7; Figure S4A in Supplementary Material). Downstream signaling cascade of common-γ-chain receptor upon IL-15 stimulation was observed, including the activation PI3K/Akt cascade (phosphorylation of 4E-BP1, leading to the disruption of its interaction with eIF4E and the activation of cap-dependent translation, and phosphorylation of p70 S6 Kinase) and the p38 MAPK pathway (Figure 2E; Figure S4B in Supplementary Material). The role of p38 MAPK, ERK1/2, and AKT pathways in the proliferation of TEMRA CD8 induced by IL-15 stimulation was then investigated using selective inhibitors (SB203580, PD98059, and LY294002, respectively). Inhibition of PI3K/Akt pathway prevents the proliferation of TEMRA and EM induced by IL-15 (Figure 2F). Of interest, the p38 MAPK and ERK1/2 pathway are required for IL-15-induced proliferation for TEMRA cells but not for EM cells. Of note, no difference in apoptotic rate was observed with the tested inhibitors (Figure S4C in Supplementary Material). Altogether, these data highlight the important differences in the required signaling events induced by IL-15 and the resulting involvement of PI3K/Akt, p38 MAPK, and ERK1/2 pathways in proliferative activity in TEMRA and EM CD8 T cells.

### TEMRA CD8 T Cells Exhibit Functional Mitochondria

**Figure 3:**
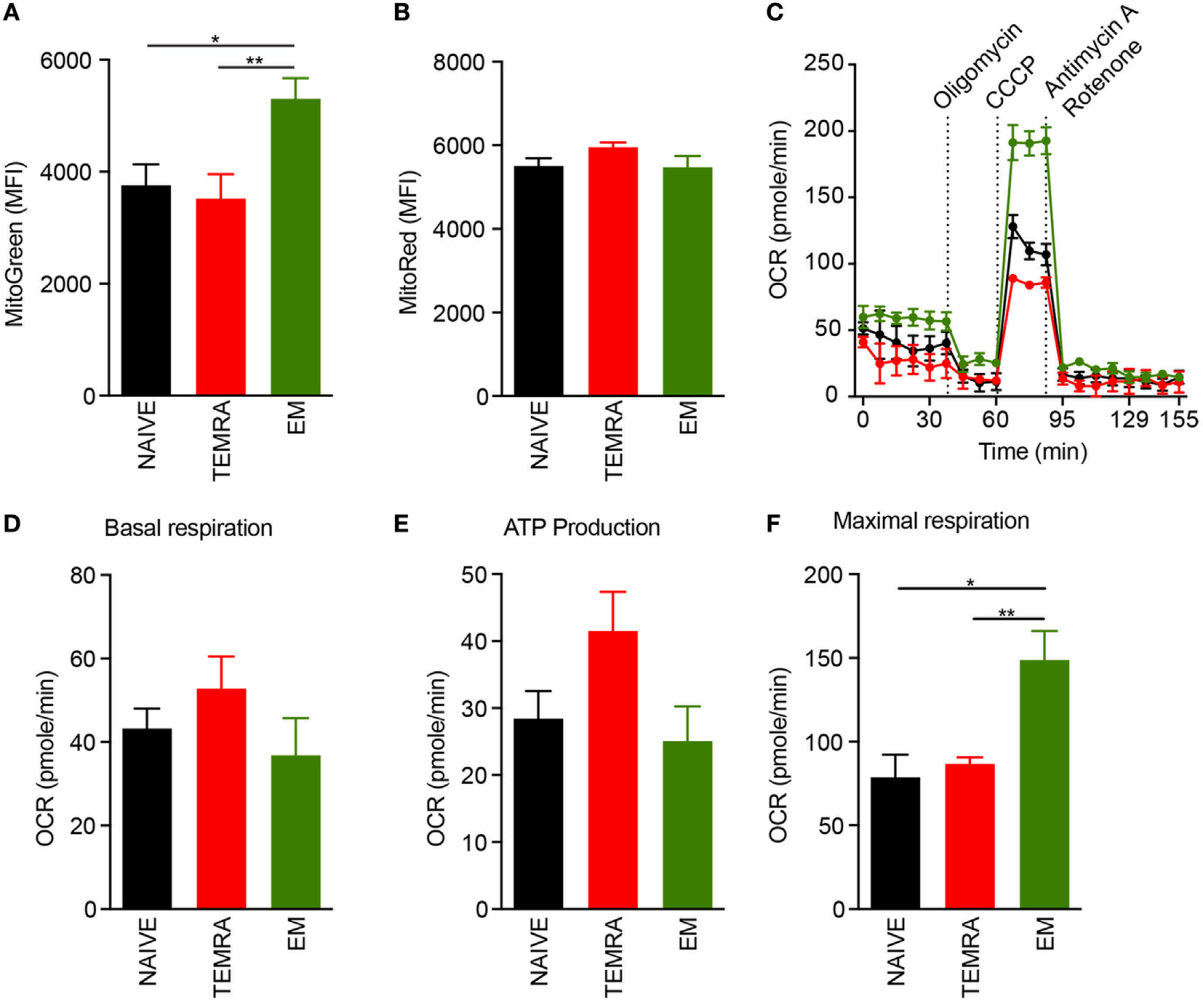
TEMRA CD8 T cells exhibit polarized and functional mitochondria. **(A,B)** Mitochondrial load **(A)** and mitochondrial membrane potential **(B)** of each CD8 T cell subsets were assessed according to mean fluorescence intensity of Mitotracker Green and Red, respectively. Oxygen consumption rate (OCR) of purified CD8 T cell subsets was measured before and after sequential addition of metabolic stress drugs. **(C)** OCR profile of one out of 5 healthy volunteers. **(D)** OCR under resting settings of purified CD8 T cell subsets. **(E)** Oxygen consumption devoted to ATP production by mitochondria was assessed by comparing OCR before and after addition of oligomycin, an inhibitor of ATP synthase, to purified CD8 T cell subsets. **(F)** Maximal respiration of purified CD8 T cell subsets after addition of CCCP. Bars indicate mean ± SEM of pooled data (*n* = 5–13). Comparison between the three CD8 subsets was performed using Kruskal–Wallis test followed by a Dunn’s Multiple Comparison Test (*p < 0.05, **p < 0.01).

The responsiveness of TEMRA CD8 T cells to IL-15, including their vigorous proliferation (Figure 2), hints at the involvement of metabolic controls regarding their immune function. In steady-state, we found that TEMRA and NAÏVE CD8 exhibit a lower mitochondrial content than EM CD8, even though the energization of mitochondria was similar, as shown with the MMP using the voltage-dependent MitoTracker probe (Figures 3A,B). Mitochondrial respiration was assessed under steady-state in purified NAIVE, TEMRA, and EM CD8 T cells using Seahorse technology. Basal respiration was similar across the different CD8 subsets (Figures 3C,D). Mitochondrial coupling efficiency was then measured after injection of oligomycin, an inhibitor of ATP synthase. All cell subsets, including TEMRA cells, exhibited well-coupled mitochondria with roughly 70% of mitochondrial respiration devoted to ATP production (Figure 3E). The addition of CCCP, a protonophore which increases mitochondrial respiration to its maximal rate, resulted in an increase in OCR in all CD8 subsets (Figures 3C,F). However, EM exhibited a different metabolism with increased respiratory reserve (Figure 3F). Collectively, these results show that TEMRA CD8 T cells exhibit well-functioning mitochondria under steady-state.

### Metabolic Profile of Resting TEMRA CD8 T Cells Reveals a Large Pool of Preformed ATP and the Activation Mechanisms of the Metabolic Pathways

**Figure 4:**
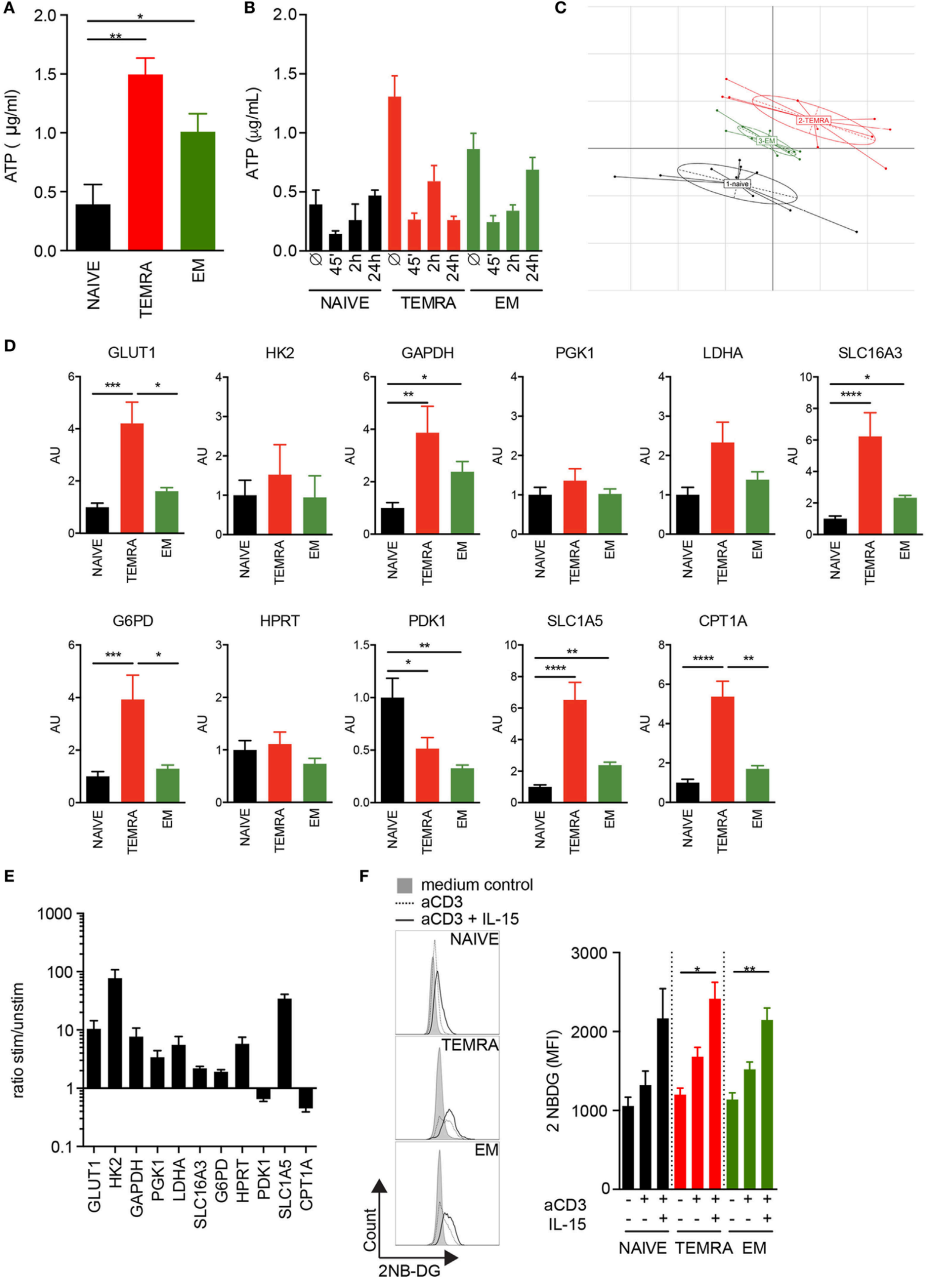
Metabolic profile of resting TEMRA CD8 T cells reveals a large pool of preformed ATP and the activation mechanism of the metabolic pathways. ATP was quantified in freshly purified CD8 T cell subsets **(A)** or after 45′, 2 h, and 24 h of stimulation with plate-bound anti-CD3 (2 µg/mL) and IL-15 (10 ng/mL) **(B)**. Bars indicate mean ± SEM of pooled data (*n* = 6–10). **(C)** Principal Component Analysis of 11 metabolic genes expressed by CD8 subsets purified from 9 to 10 healthy volunteers (HV). **(D)** Relative mRNA expression levels of genes involved in glycolysis, glutaminolysis, pentose phosphate pathway, and lipid oxidation assessed in NAIVE, TEMRA, and effector memory (EM) CD8 purified 9 to 10 HV. **(E)** Ratio of relative mRNA expression of metabolic genes expressed by TEMRA after and before stimulation for 48 h with plate-bound anti-CD3 (2 µg/mL) and IL-15 (10 ng/mL). **(F)** Uptake of 2-NBDG by purified CD8 T cell subsets stimulated for 24 h with plate-bound anti-CD3 (2 µg/mL) and IL-15 (10 ng/mL). Representative flow data are shown and bars indicate mean ± SEM of pooled data (*n* = 5–9). Kruskal–Wallis test followed by a Dunn’s Multiple Comparison Test **(A,D)** and Mann–Whitney test (F) were performed (*p < 0.05, **p < 0.01, ***p < 0.001, ****p < 0.0001).

The rapid response of TEMRA CD8 T cells to IL-15 stimulation could reflect a bioenergetic advantage of TEMRA cells. ATP content was first quantified in resting CD8 T cell subsets. TEMRA CD8 cells exhibit a high pool of available ATP, similar to that of EM cells, whereas the ATP content of Naive CD8 cells was low (Figure 4A). TCR stimulation combined with IL-15 results in a rapid consumption of the ATP reservoir, observed as early as 45′ post-stimulation, in a manner similar to EM cells (Figure 4B). However, whereas EM CD8 cells start to reconstitute their ATP reservoir 24 h post-stimulation, TEMRA CD8 cells fail to do so (Figure 4B). Thus, the high ATP level available in TEMRA and EM CD8 T cells allows for a rapid immune response as shown by the rapid consumption of the initial ATP pool. However, the delay in the ability between EM and TEMRA cells to reconstitute at least their initial energy supply within 24 h was observed, suggesting distinct metabolic features between TEMRA and EM cells.

In order to gain insight into the mechanism responsible for the difference of the quiescent ATP content observed across the CD8 subsets, the expression levels of candidate genes implicated in glycolysis, glutaminolysis, and fatty acid oxidation (FAO) were measured. Principal Component Analysis of genes expressed by resting CD8 subsets showed that TEMRA differs from EM and NAÏVE (Figure 4C). Focus on key genes involved in the various steps of the glycolytic pathway showed that TEMRA CD8 exhibits higher levels of the glucose transporter *GLUT1* as compared to NAÏVE and EM CD8 (Figure 4D). TEMRA CD8 also exhibit higher levels of expression of *GAPDH, LDHA*, and *SLC16A3* as compared to naïve and EM cells (Figure 4D). These three enzymes are involved in the glycolysis process and the export of lactate, final product of glycolysis, thus suggesting higher rate of glycolysis in all three CD8 subsets. Interestingly, other key enzymes involved in various metabolic processes, such as the pentose phosphate pathway (glucose-6-phosphate dehydrogenase, *G6PD*), glutaminolysis (glutamine transporter, *SLC1A5*), and FAO (carnitine palmitoyl transferase, *CPT1A*), were also upregulated in TEMRA CD8 cells when compared to NAÏVE and EM (Figure 4D). We then questioned how TEMRA CD8 cells adjust their metabolism after stimulation by TCR and IL-15 (Figure 4E). Mitochondrial fatty oxidation was downregulated after 48 h of stimulation as shown by the decrease of *CPT1A* expression. However, all other processes, glycolysis, glutaminolysis, and PPP, were significantly upregulated in activated TEMRA CD8 cells (Figure 4E). Furthermore, both cytosolic conversion of pyruvate to lactate through LDH and mitochondrial oxidation of pyruvate through Pyruvate Dehydrogenase were increased after stimulation, as shown, respectively, by the increased expression of LDH and the decreased expression of PDK responsible of PDH inhibition. These results suggest that purified TEMRA CD8 cells increase their uptake of glucose after stimulation to satisfy their bioenergetic needs. Indeed, an increase in glucose uptake in TEMRA CD8 T cells was observed 24 h after IL-15 stimulation (Figure 4F). Similar results were observed in Naive and EM cells.

Collectively, these results show that in the absence of *ex vivo* stimulation, TEMRA cells are metabolically more active than Naive and EM CD8 cells, which is in agreement with their high ATP reservoir. Moreover, upon stimulation, TEMRA CD8 T cells increase several metabolic pathways, such as glycolysis, glutaminolysis, and PPP, to fulfill their metabolic requirements.

### TEMRA CD8 T Cells Adapt Their Metabolism to Immunostimulation

**Figure 5:**
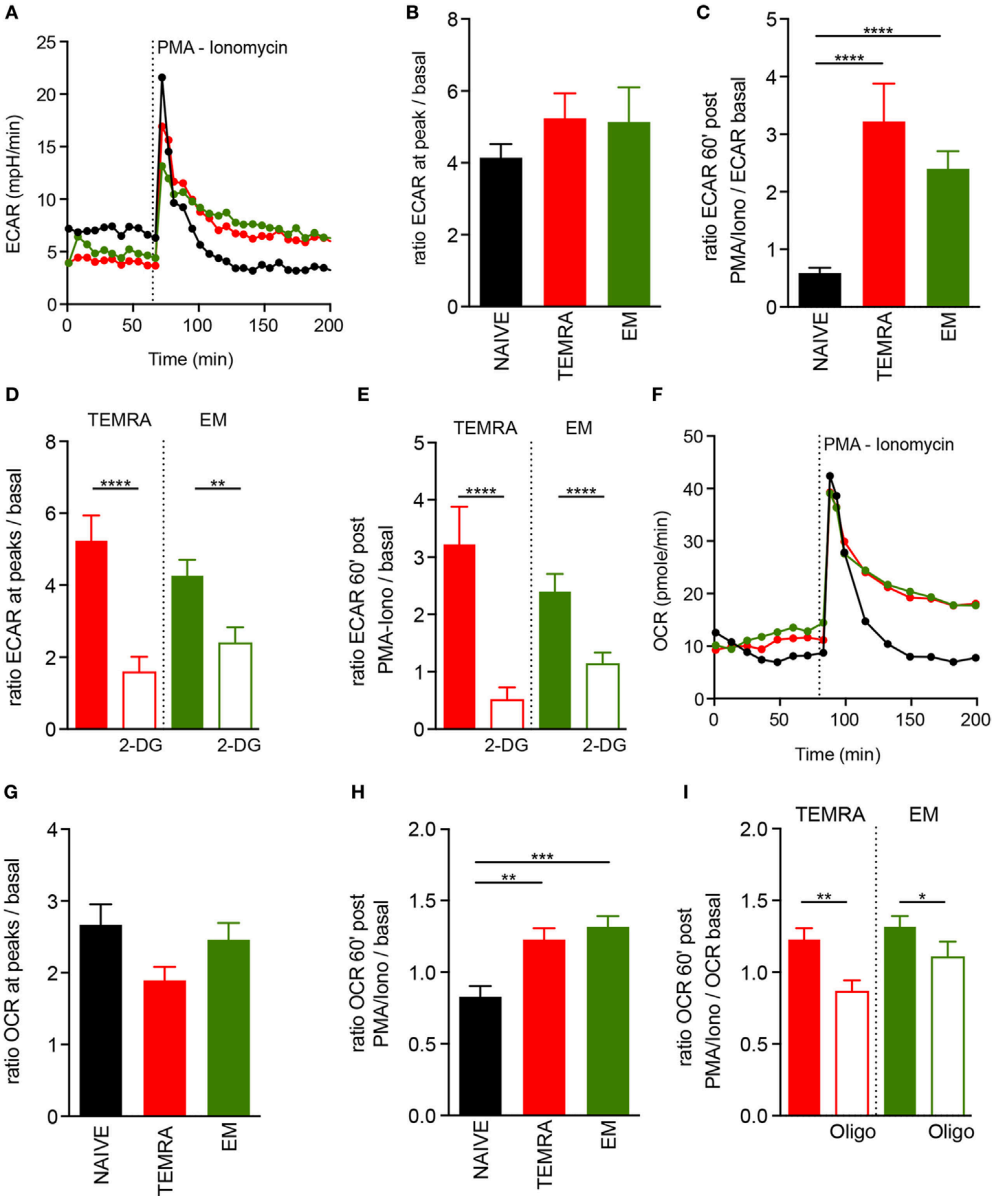
Fitness of metabolic adaptation of TEMRA CD8 T cells upon activation. **(A–C)** Extracellular acidification rate (ECAR) profile of one out of 14 healthy volunteer (HV) is shown [**(A)** NAIVE, black; TEMRA, green; effector memory (EM), red]. ECAR modification within each CD8 subset induced by PMA/Iono stimulation was assessed according to the ratio of extracellular acidification rate (ECAR) after/before drug injection [*n* = 14; **(B)**] and 60′ after/before drug injection [*n* = 14; **(C)**]. Glycolysis was inhibited by the provision of 2-DG before PMA/Iono stimulation and a similar readout was used [*n* = 11–14; **(D,E)**]. Oxygen consumption rate (OCR) of purified CD8 T cell subsets (NAIVE, black; TEMRA, green; EM, red) upon PMA/Iono stimulation was assessed, and 1 of 14 HV is shown **(F)**. OCR modification within each CD8 subset induced by PMA/Iono stimulation was assessed according to the ratio of OCR after/before drug injection [*n* = 14; **(G)**] and 60′ after/before drug injection [*n* = 14; **(H)**]. Mitochondrial respiration was inhibited by provision of oligomycin before PMA/Iono stimulation and similar readout was used [*n* = 11–14; **(I)**]. Bars indicate mean ± SEM of pooled data (*n* = 11–14). Kruskal–Wallis test followed by a Dunn’s Multiple Comparison Test **(B,C,G,H)** and Mann–Whitney test **(D,E,I)** were performed (*p < 0.05, **p < 0.01, ***p < 0.001, ****p < 0.0001).

The ability to switch rapidly from mitochondrial respiration to glycolysis and to sustain a glycolytic rate after stimulation has been shown to be a key feature of memory cells (19). ECAR was used as an indicator of glycolysis in TEMRA cells in steady-state and upon polyclonal stimulation. All CD8 T cell subsets had similar basal glycolytic profiles and displayed a great increased in ECAR within minutes following polyclonal stimulation (Figures 5A,B). However, as expected, increased ECAR was sustained after polyclonal stimulation in EM CD8 T cells but not in Naive cells (19). Interestingly, TEMRA exhibited a similar ECAR profile after stimulation as EM cells (Figures 5A,C). The addition of 2-deoxyglucose (2-DG), which blocks the first step of the glycolytic pathway, prevented both the immediate and the sustained rise of ECAR (Figures 5D,E). Collectively, these data show that TEMRA CD8 T cells have the same ability as EM CD8 T cells to rapidly engage glycolysis for a prolonged period upon polyclonal stimulation.

We then assessed whether mitochondria were involved in the T cells metabolic response upon polyclonal stimulation. Polyclonal stimulation of TEMRA CD8 T cells resulted in an immediate and rapid increase of OCR (Figures 5F,G), with a similar magnitude to those of NAIVE and EM CD8 T cells. However, like EM CD8 T cells, TEMRA CD8 T cells sustained an increased OCR overtime, whereas the mitochondrial respiration of NAIVE CD8 T cells returned rapidly to basal level (Figure 5H). As expected, the provision of oligomycin prevented the sustained increase in OCR (Figure 5I). Together, these data showed that TEMRA CD8 T cells have the same ability as EM CD8 T cells to rapidly adapt their metabolism to polyclonal stimulation by increasing both mitochondrial respiration and glycolysis for a prolonged period.

### Immune Function of TEMRA CD8 Can Be Abrogated by the Interference with Glycolysis, Glutaminolysis, and Mitochondria Respiration

**Figure 6:**
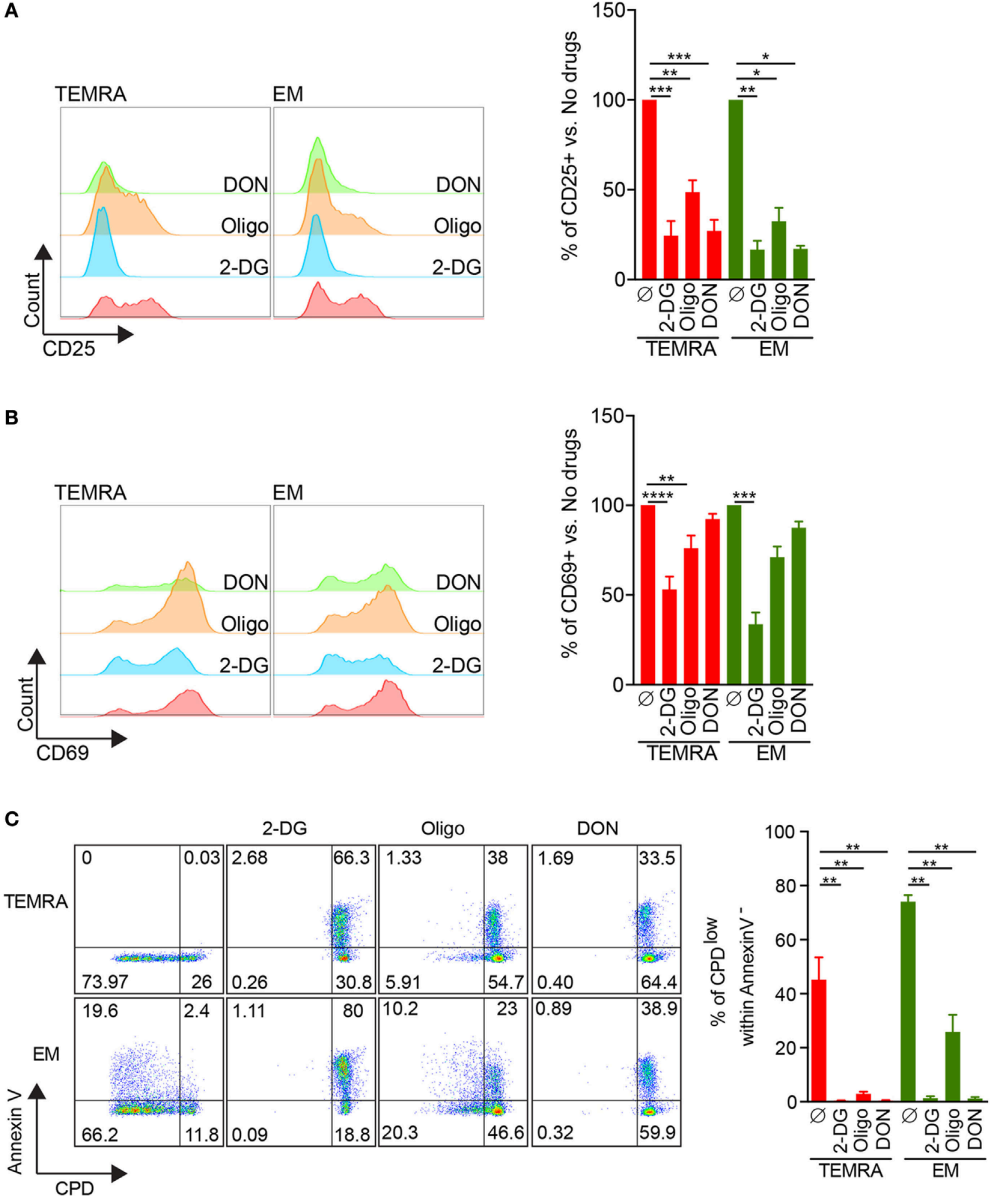
2-DG, DON, or oligomycin potently prevent IL-15-induced activation of TEMRA CD8. **(A,B)** Expression of CD25 **(A)** or CD69 **(B)** by TEMRA and effector memory (EM) CD8 T cells stimulated with plate-bound anti-CD3 (2 µg/mL) and IL-15 (10 ng/mL) for 24 h in the presence of 2-DG, oligomycin, or DON. Representative flow data are shown and bars indicate mean ± SEM of pooled data (*n* = 5–7). **(C)** Percentage of CPDeFluor405^low^ cells within Annexin V^−^ cells after 5 days of culture of purified CD8 subsets with plate-bound anti-CD3 (2 µg/mL) and IL-15 (10 ng/mL) in the presence of medium control or selective inhibitors (2-DG, oligomycin, DON). Representative flow data are shown and bars indicate mean ± SEM of pooled data (*n* = 5–7). Kruskal–Wallis test followed by a Dunn’s Multiple Comparison Test was performed (*p < 0.05, **p < 0.01, ***p < 0.001, ****p < 0.0001).

The ability to control the immune response through metabolic interferences has gained a growing interest in the literature, and it has been shown that selected or combined inhibitions of glycolysis with 2-DG, glutaminolysis with DON, and OXPHOS using oligomycin or metformin can alter T cell immune function (13–17). Thus, we investigated whether the immune function of TEMRA CD8 can be controlled using metabolic specific inhibitors (2DG, DON, and oligomycin). IL-15-induced expression of CD25 by TEMRA cells could be efficiently inhibited when glycolysis or glutaminolysis were inhibited (inhibition greater than 75%; Figure 6A). Oligomycin was not as potent to prevent the expression of CD25 on TEMRA CD8 cells (52% of inhibition; Figure 6A). In contrast, only glycolysis inhibition could prevent the expression of CD69 induced by IL-15 stimulation (47, 24, and 8% of inhibition when 2-DG, oligomycin, and DON were used, respectively; Figure 6B). No difference between TEMRA and EM CD8 cells was noticed as for the efficacy of metabolic interferences to prevent CD25 and CD69 expression (Figures 6A,B). Given that CD69 is only an early marker of proliferation, we tested whether prolonged inhibition of metabolic pathways could prevent the proliferation of TEMRA CD8 induced by IL-15 stimulation. To take into account the potential toxicity of prolonged inhibition of metabolic processes, the analysis of the proliferation was restricted to Annexin V^-^ cells. The inhibition of glycolysis with 2-DG, glutaminolysis with DON, and OXPHOS using oligomycin was able to abrogate the proliferation of TEMRA cells induced by IL-15 stimulation (Figure 6C) with a similar efficacy across the three drugs. Of interest, whereas 2-DG and DON blunt the proliferation of EM, oligomycin only partially controlled the IL-15-induced proliferation (Figure 6C). Therefore, we have determined that blocking glycolysis, glutaminolysis, and mitochondrial respiration can effectively counteract and inhibit the activation and proliferative effect of IL-15 stimulation on TEMRA CD8 T cells. Moreover, inhibition of mitochondrial respiration prevents the proliferation of TEMRA CD8 while relatively sparing the function of EM CD8.

### Inhibition of Glycolysis and Glutaminolysis Efficiently Prevent the Inflammation of the Endothelium Induced by TEMRA CD8 from KT Recipients

**Figure 7:**
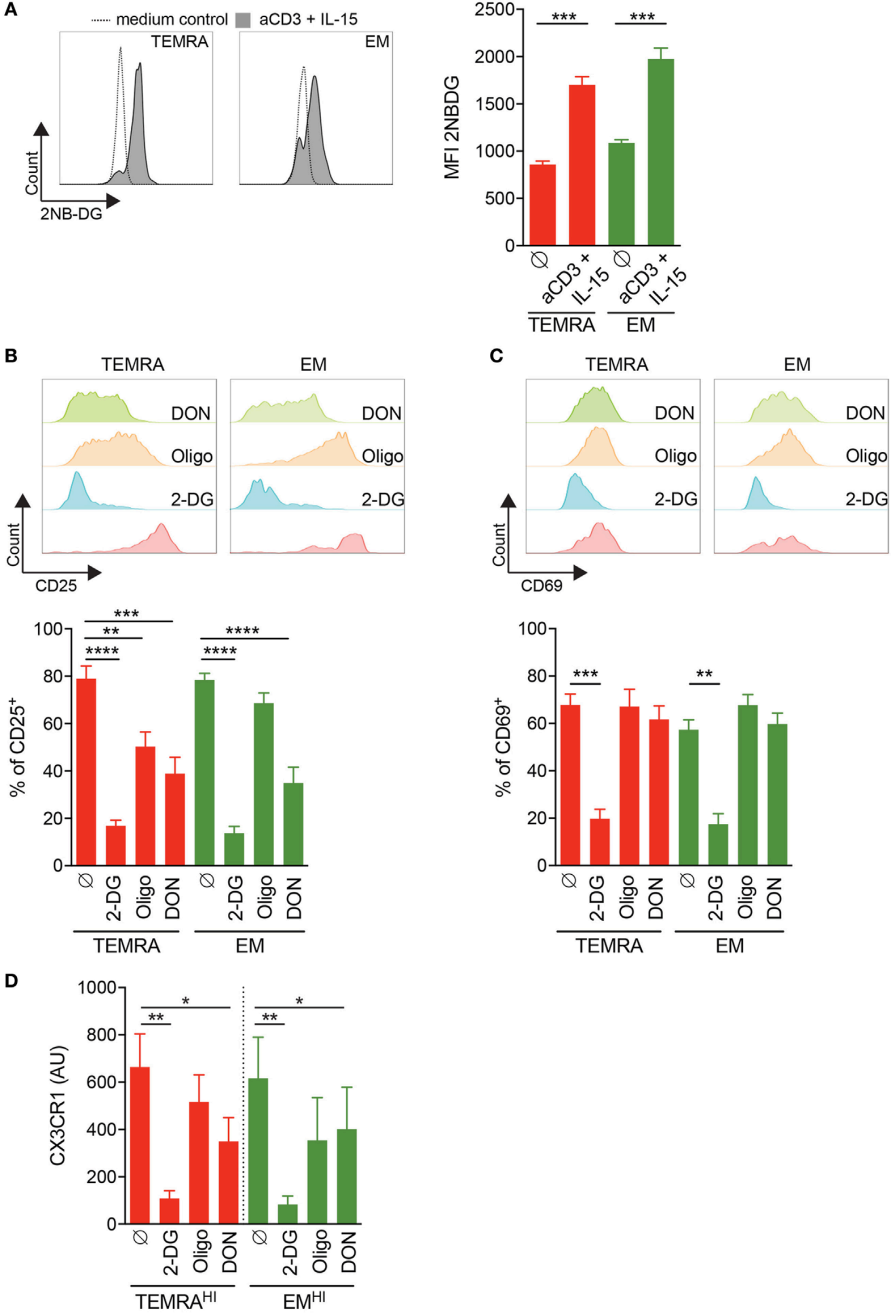
Inhibition of IL-15 induced activation of TEMRA CD8 T cells from kidney-transplant (KT) recipients by interferences with glycolysis or glutaminolysis. **(A)** Uptake of 2-NBDG by CD8 T cell subsets purified from KT recipients stimulated for 24 h with plate-bound anti-CD3 (2 µg/mL) and IL-15 (10 ng/mL). Representative flow data are shown and bars indicate mean ± SEM of pooled data (*n* = 9). **(B,C)** Expression of CD25 or CD69 by CD8 T cell subsets purified from KT recipients stimulated for 24 h with plate-bound anti-CD3 (2 µg/mL) and IL-15 (10 ng/mL) in the presence of 2-DG, oligomycin, DON, or control medium. Representative flow data are shown and bars indicate mean ± SEM of pooled data (*n* = 10–12). **(D)** 2-DG, oligomycin, or DON was added during the overnight stimulation of CD8 T cell subsets from KT before the coculture of human umbilical vein endothelial cell with supernatant from IL-15-activated CD8 T cell subsets. Bars indicate mean ± SEM of pooled data (*n* = 4–6). Mann–Whitney test or Kruskal–Wallis test followed by a Dunn’s Multiple Comparison Test were performed (*p < 0.05, **p < 0.01, ***p < 0.001, ****p < 0.0001).

Finally, we assessed whether in a context of chronic stimulation pro-inflammatory TEMRA CD8 T cells could be also controlled by blocking glycolysis, glutaminolysis, and mitochondrial respiration. First, we tested the ability of CD8 subsets from KT recipients to increase the uptake of glucose upon IL-15 stimulation. After 24 h of stimulation with plate-bound anti-CD3 and IL-15, the uptake of 2-NBDG by purified CD8 T subsets were measured (Figure 7A). TEMRA CD8 cells from KT recipients increase the uptake of glucose, demonstrating their responsiveness to IL-15 stimulation and their ability to modify their nutriment uptake to satisfy their bioenergetics need.

We then assessed how TEMRA from KT recipients relies on glycolysis, glutaminolysis, and OXPHOS to upregulate the expression of CD25 and CD69. As observed in HV (Figures 6A,B), the activation of TEMRA CD8 from KT recipients relies on all three metabolic pathways as the expression of CD25 induced by IL-15 is dampen in the presence of 2-DG, oligomycin, and DON (Figure 7B). Nevertheless, inhibition of glycolysis by 2-DG was the most effective drugs effective in preventing the activation of TEMRA cells induced by IL-15. Of interest, in KT recipients, TEMRA and not EM CD8 were susceptible to OXPHOS inhibition by olygomycin (Figure 7B). CD69 upregulation on TEMRA or EM CD8 was only prevented by the provision of 2-DG (Figure 7C). DON and oligomycin failed to prevent the upregulation of CD69 by TEMRA or EM from KT recipients, as also observed in HV (Figures 7C and 6B). Collectively, we have demonstrated that the early activation of TEMRA CD8 from KT recipients can be controlled by interfering with glycolysis, glutaminolysis, or mitochondrial respiration.

Finally, we questioned the ability of 2-DG, oligomycin, and DON to prevent the CD8 cell-mediated inflammation of endothelial cells by blocking the metabolic pathway during the activation of CD8 T cell from KT recipients (Figure 7D). Inhibition of glycolysis or glutaminolysis prevented the damage to HUVEC induced by TEMRA^HI^ and EM^HI^ CD8 T cells from KT recipients with more than 50% of reduction in the expression of CX3CL1. Inhibition of glycolysis by 2-DG was more effective at preventing the upregulation of CX3CL1 by HUVEC compared to the inhibition of glutaminolysis by DON (85 to 90% and 51 to 53% of inhibition when 2-DG or DON was added to TEMRA^HI^ or EM^HI^ CD8 T cells from KT recipients, respectively; Figure 7D). In contrast, the inhibition of mitochondrial respiration by oligomycin resulted in a small decrease of CX3CL1 by HUVEC (22 to 35% of inhibition when oligomycin was added to TEMRA^HI^ or EM^HI^ CD8 T cells from KT recipients, respectively; Figure 7C). Overall, our data show that the pathogenic function of TEMRA CD8 from KT recipients can be blunted by interfering with the glycolysis and glutaminolysis pathways.

## Discussion

Reports in the literature support the involvement of TEMRA CD8 in pathogenic processes ranging from autoimmunity to allo-transplantation (1–3) but the identification of factors regulating their expansion and their function remain ill-defined. Moreover, the lack of appropriate stimuli to activate TEMRA CD8 has precluded the screening of therapeutic drugs. In this report, we undertook a side-by-side comparison of immune-metabolic properties of TEMRA and EM CD8 in HV and in KT, and we highlighted unique features of TEMRA CD8. We provide evidences that IL-15 is a potent activator of TEMRA CD8 regardless the immune challenge of the individual. Stimulating TEMRA CD8 cells with IL-15 induces an early and rapid activation, as exemplified by the upregulation of CD25 and CD69, and with a faster kinetic as compared to EM CD8. Of note, the chronic stimulation of the immune system in the context of immunosuppression does not preclude the capacity of TEMRA CD8 to be activated by IL-15. We also show that, upon IL-15 stimulation, TEMRA CD8 from KT promote inflammation by inducing the expression of CX3CL1 by endothelial cells in an IFN-γ- and TNF-α-dependent manner. Finally, the inhibition of glycolysis is the most effective strategy to control the pathogenicity of TEMRA CD8 in KT.

The pleiotropic role of IL-15 has been well characterized. Basal levels of IL-15 favor a slow and steady rate of turnover of memory CD8 T cells (9, 29). Under inflammatory conditions, such as viral infection, type I IFN drives the secretion of IL-15 which subsequently favors the rapid division of memory CD8 T cells following an antigen encounter and enhances their protective capacity against viral infection (12). IL-15 favors the migration of memory CD8 T cells toward inflamed tissues (11) and supports the proliferation and the survival of CCR7^−^ memory CD8 T cell pool (30). The CCR7^−^ memory CD8 include EM and TEMRA with a heterogeneous frequency across HV and in patients with strong immune stimulation such as kidney transplantation (25). By investigating the reactivity of TEMRA and EM CD8 and by characterizing their metabolic profiles, we aimed to assess whether TEMRA and EM CD8 respond in similar manner to IL-15 stimulation. Of note, in the literature, very few reports have characterized the metabolic profiles of TEMRA CD8 (20, 21) and none have interrogated the regulation of their metabolism by IL-15. We show that TEMRA and EM CD8 exhibit an activated metabolic profile upon IL-15 stimulation. This is characterized by an activated composite-metabolism including glycolysis, glutaminolysis, and FAO, a high load of preformed ATP pool that can be rapidly mobilized. TEMRA CD8 can adapt their metabolism to the ATP-consuming processes needed to secrete pro-inflammatory cytokines and proliferate, as shown by the increase in glucose import and the rapid and sustained increase in glycolysis and mitochondrial respiration when strong polyclonal stimulus was used. Considering the high amounts of transcriptional factors T-bet and Eomes associated with a high expression of cytotoxic molecules and pro-inflammatory cytokines, and the restricted TCR Vβ repertoire, we propose that TEMRA CD8, defined as CD45RA^+^CD28^−^CD8^+^, are a subset of EM CD8 with dedicated role in immune-surveillance. TEMRA CD8 can mount a rapid response to stimulation that may be beneficial in the context of HV but deleterious in the context of chronic stimulation such as allo-transplantation (3, 31) and autoimmune diseases (1, 2). The design of innovative strategies to control the immune functions of TEMRA CD8 is thus needed.

The use of metabolic inhibitors to control the immune response has become popular in the recent years. The changes in intracellular metabolic pathways alter the immune function (32) and numerous reports have shown the feasibility of such approaches in animal models, including lupus (13), tumor vaccination (14), hematopoietic stem cell transplantation (15, 16), and heart and skin transplantation (17). The control of the immune function of CD8, including TEMRA CD8, from KT by the selective inhibition of metabolic pathways remains an unresolved question. In this report, we show that the inhibition of glycolysis, glutaminolysis, and mitochondrial respiration pathways was effective at preventing proliferation of TEMRA CD8 derived from HV. Drug screening using early marker of activation (CD25 and CD69) revealed a heterogeneity in the inhibition efficacy ranging from high inhibition with 2-DG to low inhibition with oligomycin. These data strengthen the need to combine multiple readout of immune markers. Interestingly, our findings extended to TEMRA CD8 purified from KT. The immune system of KT has undergone a strong immune challenge under a suboptimal setting due to the daily administration of immune-suppressive drugs. This could potentially modulate the metabolic fitness of the TEMRA CD8. Activating TEMRA CD8 from KT with IL-15 resulted in an increase in glucose uptake, the acquisition of early activation markers and the release of pro-inflammatory cytokines (IFN-γ and TNF-α) which trigger endothelium inflammation. As for HV, inhibition of glycolysis was the most effective strategy to control TEMRA CD8. IL-15-induced upregulation of CD25 and CD69 was efficiently inhibited by 2-DG as well as the inflammation of endothelial cells. The modulation of glutaminolysis or mitochondrial respiration was less effective at preventing the inflammation of the endothelium. Collectively, these results identified the involvement of TEMRA CD8 in the inflammation of the endothelium by enhancing the recruitment of CX3CR1-expressing immune cells and that specifically targeting the glycolytic pathway and to a lesser extent glutaminolysis pathway is highly effective at controlling their pathogenicity.

In conclusion, we have identified a key role of IL-15 in the activation of TEMRA CD8 in resting and immune systems with strong immune stimulation (i.e., kidney transplantation). With their functional mitochondria, large reserves of ATP, and their rapid metabolic adaptation, TEMRA CD8 are able to rapidly and efficiently engage their effector functions through pro-inflammatory cytokines and cytotoxic molecules secretion and foster inflammation by activating the endothelium. Our findings indicate that drugs that can alter glycolysis or glutaminolysis could be promising treatments for diminishing any deleterious effects of TEMRA CD8 on KT and more broadly in immune settings in which high frequency of TEMRA CD8 have been observed.

## Ethics Statement

All subjects gave written informed consent in accordance with the Declaration of Helsinki. HV were enrolled by the Etablissement Français du Sang (EFS, Nantes, France) within the context of a research contract. A convention has been signed between our laboratory (CRTI—INSERM UMR 1064) and the blood bank (Etablissement Français du Sang Pays de La Loire) and approval of an ethical committee was thus not necessary. The University Hospital Ethical Committee and the Committee for the Protection of Patients from Biological Risks approved the study for patients. The biological samples and data are gathered in accordance with French Law, more specifically with “Bioethical law” of August 6, 2004, Act no. 78-17 of January 6, 1978 on data processing, data, files, and individual liberties, with the European regulation: Directive 2004/23/EC of European Parliament and of the council of March 31, 2004, on setting, standards of quality and safety of donation, procurement, testing, processing, preservation, storage, and distribution of human tissue and cells, and with Directive 95/46/EC on the protection of individuals with regard to the processing of personal data and on the free movement of such data.

## Author Contributions

GT, T-MD-N, and MY performed and analyzed the experiments. BN and AG performed the experiments. LJ helped to write the manuscript. AC and SB performed the HUVEC-related experiments. RD performed statistical analysis. MC performed the TCR Vb repertoire assay. PG, MG, and D-AL provided human samples. ND designed, performed, and analyzed experiments and, together with CH and Sophie B, wrote the manuscript. All authors contributed to general design and discussion of the project and reviewed and approved the manuscript.

## Conflict of Interest Statement

The authors declare that the research was conducted in the absence of any commercial or financial relationships that could be construed as a potential conflict of interest.

## Supplementary Material

Figure S1 Strategy of gating and sorting of CD8 subsets. After gating on CD3^+^CD8^+^ cells, CD45RA and CD28 were used to identify effector memory (EM) (CD45RA-CD28^+^; I), NAÏVE (CD45RA^+^CD28^+^; II), and TEMRA (CD45RA^+^CD28^−^; III). As expected, CCR7 was homogenously expressed by NAÏVE CD8 T cells whereas no expression was detected on the surface of EM and TEMRA.

Figure S2 IL-15 stimulation enhances the effector function of TEMRA CD8 cells. **(A)** NAIVE, TEMRA, and effector memory (EM) CD8 T cells were FACS-sorted from seven healthy donors and expression of the mentioned transcripts was determined by qPCR. Results were normalized to 18S and data are shown in arbitrary unit. **(B,C)** Expression of selected genes in TEMRA **(B)** and EM **(C)** after 48 h of stimulation with plate-bound aCD3 and IL-15. Kruskal–Wallis test followed by a Dunn’s Multiple Comparison Test **(A)** or Mann–Whitney test **(B,C)** were performed (**p* < 0.05, ***p* < 0.01, ****p* < 0.001).

Figure S3 TEMRA CD8 cells exhibit an accumulation of selected T cell clones. TCR Vβ repertoire of purified CD8 T cell subsets from five healthy volunteers was studied by CDR3 spectratyping. **(A)** Overview of the TCR Vβ repertoire usage in NAÏVE, TEMRA, and effector memory (EM) CD8 T cells. The CDR3-LD of each TCR Vβ families was analyzed in an unbiased manner and described according to a four-type nomenclature [polyclonal, polyclonal with major peak (PAPM), oligoclonal, or monoclonal (1)]. The frequency of each type of CDR3-LD was evaluated and the mean is displayed using a 10 × 10 dot plot (i.e., each dot represents 1%). **(B,C)** TCR Vβ families of NAÏVE, TEMRA, and EM CD8 T cell were compared to the corresponding families of the unbiased reference sample (commercially available pool of human thymus). The comparisons were performed as described in Section “Materials and Methods,” using a distance score **(B)** and a correlation coefficient **(C)**. Each dot represents a comparison for 1 Vβ between the reference sample and a CD8 subset and the mean of the 120 Vβ (24 TCR Vβ × five individuals) is shown. One-way ANOVA was performed to compare the correlations and the distances, followed by a Tukey’s multiple comparison test **(B,C)** (**p* < 0.001).

Figure S4 Signaling cascade triggered by IL-15 stimulation of purified CD8 subsets. **(A,B)** Peripheral blood mononuclear cells were stimulated for 15′ with IL-15 (10 ng/mL) or medium control and phosphorylation of pSTAT5, p70 S6 Kinase (pT389), 4E-BP1 (pT37/46), and p-p38 MAPK (pT180/pY182) was analyzed within NAIVE, TEMRA, and effector memory (EM) CD8 T cells. Representative flow data are shown (*n* = 8). **(C)** Percentage of Annexin-V^low^ cells after 5 days of culture of purified CD8 subsets with plate-bound anti-CD3 (2 µg/mL) and IL-15 (10 ng/mL) in the presence of medium control or selective inhibitors (LY294002, 10 µM; PD98059, 10 µM; SB203580, 10 µM). Mean ± SEM of at least four HV is shown. Wilcoxon matched-pairs signed rank test was used to test the selective inhibitors effect (medium control as reference) **p* < 0.05, ***p* < 0.01.
