# Supplementary figures and images for: IL-15 Harnesses Pro-inflammatory Function of TEMRA CD8 in Kidney-Transplant Recipients
Source: Front Immunol. 2017 Jun 30;8:778. doi: 10.3389/fimmu.2017.00778 (PMC5492498; doi:10.3389/fimmu.2017.00778)

Supplementary Figure 1

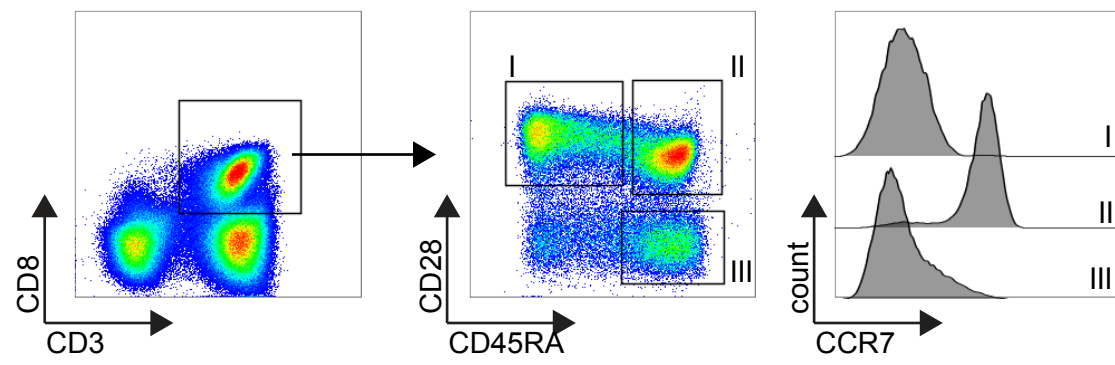

Supplement: Figure S1 — Strategy of gating and sorting of CD8 subsets. After gating on CD3+CD8+ cells, CD45RA and CD28 were used to identify effector memory (EM) (CD45RA-CD28+; I), NAÏVE (CD45RA+CD28+; II), and TEMRA (CD45RA+CD28−; III). As expected, CCR7 was homogenously expressed by NAÏVE CD8 T cells whereas no expression was detected on the surface of EM and TEMRA. [file image_1.pdf]

Supplementary Figure 2

A

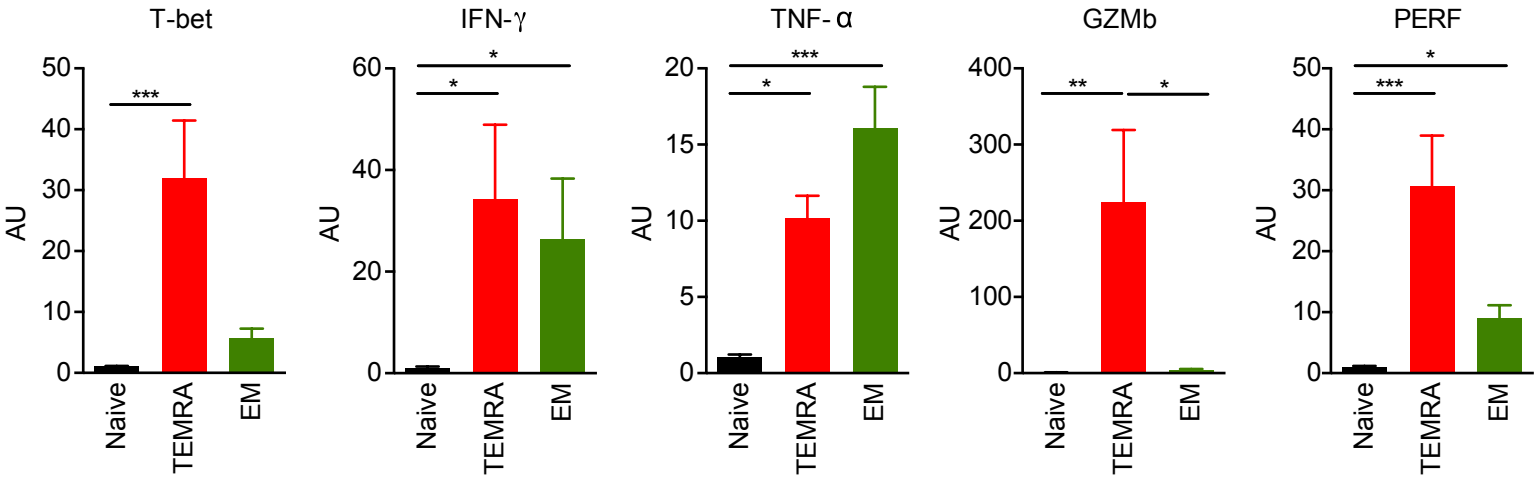

B

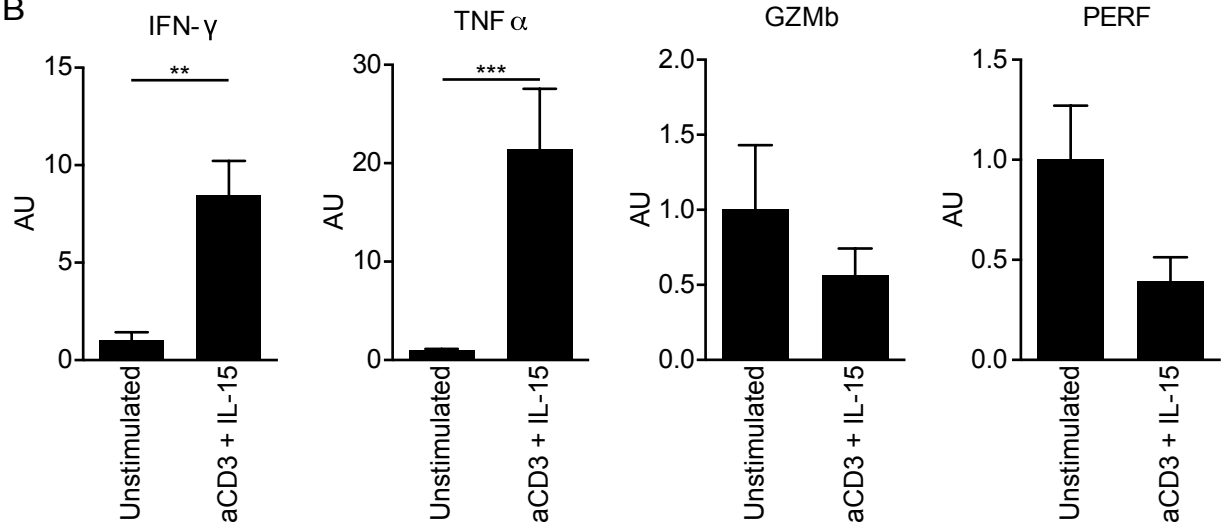

C

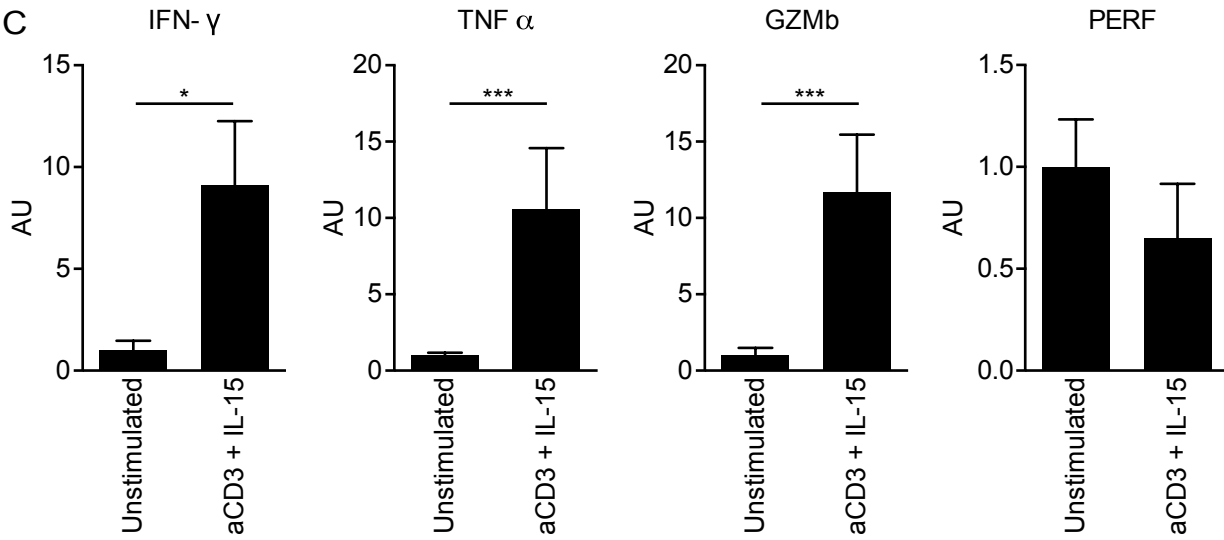

Supplement: Figure S2 — IL-15 stimulation enhances the effector function of TEMRA CD8 cells. (A) NAIVE, TEMRA, and effector memory (EM) CD8 T cells were FACS-sorted from seven healthy donors and expression of the mentioned transcripts was determined by qPCR. Results were normalized to 18S and data are shown in arbitrary unit. (B,C) Expression of selected genes in TEMRA (B) and EM (C) after 48 h of stimulation with plate-bound aCD3 and IL-15. Kruskal–Wallis test followed by a Dunn’s Multiple Comparison Test (A) or Mann–Whitney test (B,C) were performed (*p < 0.05, **p < 0.01, ***p < 0.001). [file image_2.pdf]

Supplementary Figure 3

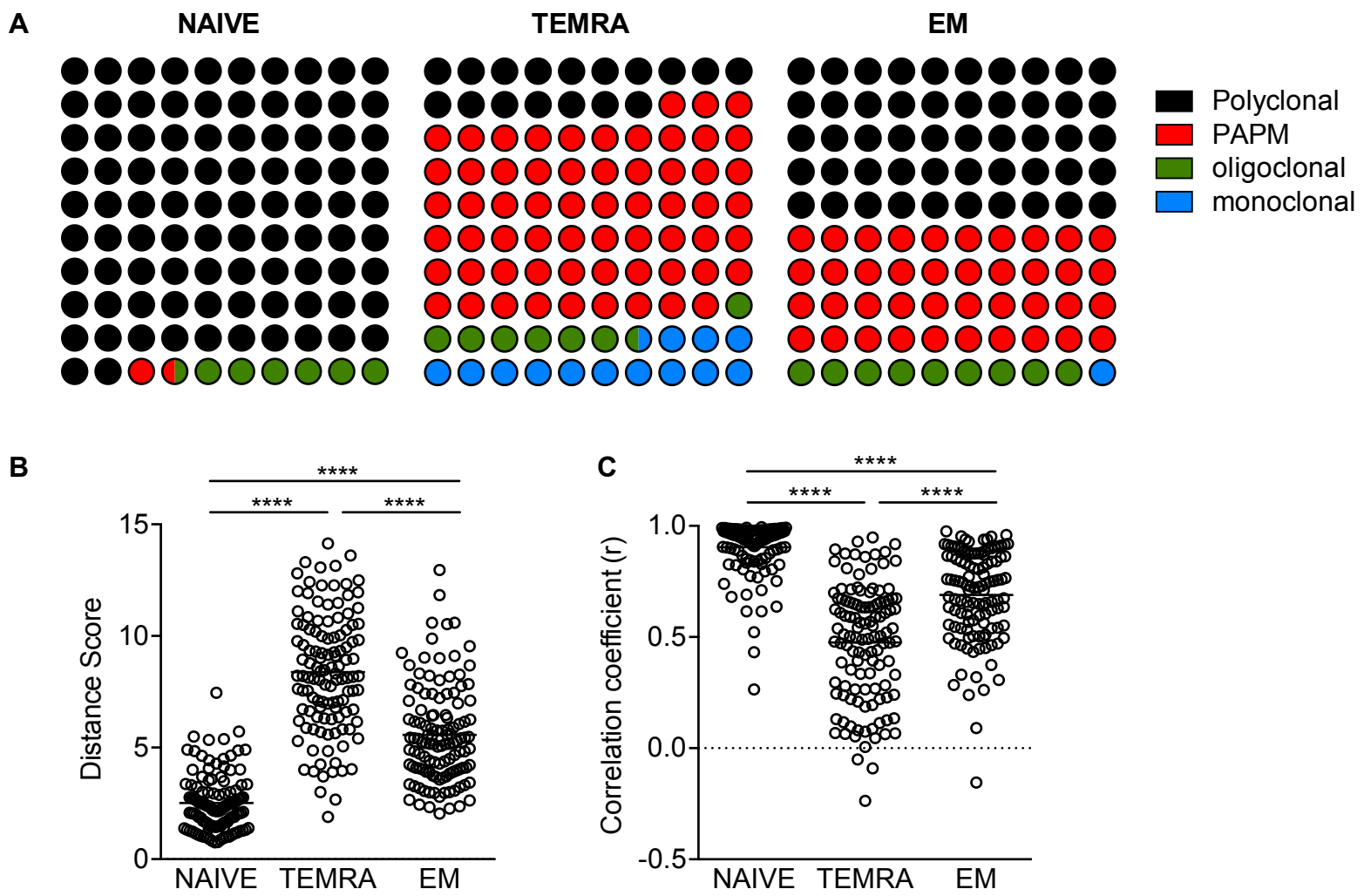

Supplement: Figure S3 — TEMRA CD8 cells exhibit an accumulation of selected T cell clones. TCR Vβ repertoire of purified CD8 T cell subsets from five healthy volunteers was studied by CDR3 spectratyping. (A) Overview of the TCR Vβ repertoire usage in NAÏVE, TEMRA, and effector memory (EM) CD8 T cells. The CDR3-LD of each TCR Vβ families was analyzed in an unbiased manner and described according to a four-type nomenclature [polyclonal, polyclonal with major peak (PAPM), oligoclonal, or monoclonal (1)]. The frequency of each type of CDR3-LD was evaluated and the mean is displayed using a 10 × 10 dot plot (i.e., each dot represents 1%). (B,C) TCR Vβ families of NAÏVE, TEMRA, and EM CD8 T cell were compared to the corresponding families of the unbiased reference sample (commercially available pool of human thymus). The comparisons were performed as described in Section “Materials and Methods,” using a distance score (B) and a correlation coefficient (C). Each dot represents a comparison for 1 Vβ between the reference sample and a CD8 subset and the mean of the 120 Vβ (24 TCR Vβ × five individuals) is shown. One-way ANOVA was performed to compare the correlations and the distances, followed by a Tukey’s multiple comparison test (B,C) (*p < 0.001). [file image_3.pdf]

Supplementary Figure 4

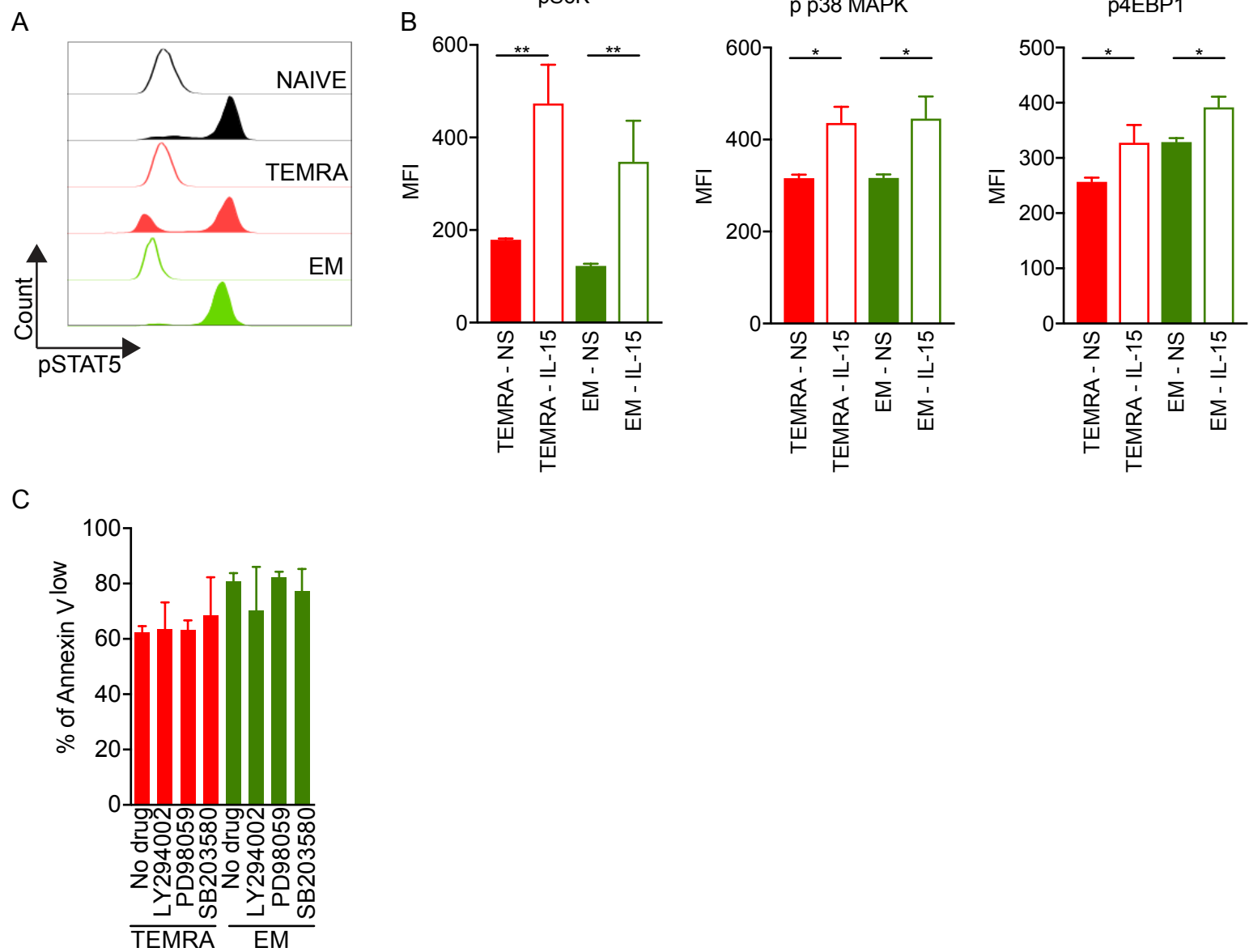

Supplement: Figure S4 — Signaling cascade triggered by IL-15 stimulation of purified CD8 subsets. (A,B) Peripheral blood mononuclear cells were stimulated for 15′ with IL-15 (10 ng/mL) or medium control and phosphorylation of pSTAT5, p70 S6 Kinase (pT389), 4E-BP1 (pT37/46), and p-p38 MAPK (pT180/pY182) was analyzed within NAIVE, TEMRA, and effector memory (EM) CD8 T cells. Representative flow data are shown (n = 8). (C) Percentage of Annexin-Vlow cells after 5 days of culture of purified CD8 subsets with plate-bound anti-CD3 (2 µg/mL) and IL-15 (10 ng/mL) in the presence of medium control or selective inhibitors (LY294002, 10 µM; PD98059, 10 µM; SB203580, 10 µM). Mean ± SEM of at least four HV is shown. Wilcoxon matched-pairs signed rank test was used to test the selective inhibitors effect (medium control as reference) *p < 0.05, **p < 0.01. [file image_4.pdf]
